# Ciprofloxacin Treatment Failure in Typhoid Fever Case, Pakistan

**DOI:** 10.3201/eid0912.030230

**Published:** 2003-12

**Authors:** Tariq Butt, Rifat Nadeem Ahmad, Abid Mahmood, Sabeen Zaidi

**Affiliations:** * Armed Forces Institute of Pathology, Rawalpindi, Pakistan

**Keywords:** *Salmonella enterica* serovar Typhi, fluoroquinolone, treatment failure

## Abstract

We report a case of ciprofloxacin treatment failure in a typhoid fever patient at a tertiary care hospital in Rawalpindi, Pakistan. This case shows not only the emergence of fluoroquinolone resistance in typhoid salmonellae but also the inadequacy of the current laboratory guidelines for detection of this resistance.

Typhoid fever is a major health concern in the developing world; >16 million new cases occur worldwide annually, resulting in approximately 600,000 deaths per year. The last two decades have seen the emergence and spread of multidrug resistance against the conventional antityphoid drugs (chloramphenicol, co-trimoxazole, and ampicillin) among the typhoid salmonellae, especially in South and Southeast Asia, including Pakistan. These developments had left fluoroquinolones as the antimicrobial agents of choice for the treatment of typhoid fever (*1*). Fluoroquinolone resistance is being reported with increasing frequency from all over the world (*1*–*5*). We report ciprofloxacin treatment failure in a case of typhoid fever.

## Case Report

A previously healthy 14-year-old boy from Rawalpindi, Pakistan, was admitted in July 2002 to Combined Military Hospital, Rawalpindi, with a 7-day history of a high fever (>38°C) and vomiting. He had relative bradycardia (heart rate 84 bpm) and a soft palpable spleen. His total leukocyte count was 3 x 10^9^/L. Malarial parasites were not seen on examination of thin and thick smears of peripheral blood. The results of a routine urinalysis and chest radiographs were normal. A blood Widal test showed a titer of 320 against “O” (somatic) antigen of *Salmonella enterica* serovar Typhi. Blood culture yielded the growth of *Salmonella* Typhi. The isolate was found to be resistant to the conventional antityphoid drugs by using modified Kirby-Bauer disk diffusion technique according to the criteria of the National Committee for Clinical Laboratory Standards (NCCLS) (*6*). The disks of antimicrobial drugs used were chloramphenicol (30 μg), co-trimoxazole (1.25/23.75 μg), ampicillin (10 μg), ciprofloxacin (5 μg), and ceftriaxone (30 μg). The isolate appeared susceptible to ciprofloxacin and ceftriaxone.

The patient was given ciprofloxacin, 500 mg, every 12 hours, orally, on admission but remained febrile after 3 days of treatment. When the blood culture report was received, and in view of the susceptibility pattern, intravenous ciprofloxacin, 200 mg every 12 hours, was administered. Despite 8 days of treatment, his fever did not resolve. The isolate was reviewed and the MIC of ciprofloxacin was determined by Kirby-Bauer broth dilution technique; it was 0.5 μg/mL; well below the NCCLS recommended break point value of 1 μg/mL (*7*). However, in light of the treatment failure with ciprofloxacin, intravenous ceftriaxone,1 g every 12 hours, was administered, and the patient responded within 3 days.

## Conclusions

This case highlights two developments: first, the increasing incidence of reduced susceptibility and resistance of typhoid salmonellae against fluoroquinolones, and second, the inadequacy of the present laboratory guidelines for detecting fluoroquinolone resistance in typhoid salmonellae. The first case of ciprofloxacin-resistant typhoid fever was reported in 1992 in the United Kingdom (*8*), and the first case of fluoroquinolone treatment failure in typhoid fever in Pakistan was reported in 1993 (*9*). Similar cases have been reported from several other countries (*1*–*5*). Selective pressure on the bacterial population by uncontrolled use of these antimicrobial drugs has likely led to the emergence of this resistance (*2*), which has been attributed to a single point mutation in the quinolone-resistance–determining region (QRDR) of the topoisomerase gene *gyrA* (*1*,*2*,*5*,*10*). However, other mechanisms such as decreased permeability and active efflux of the antimicrobial agent may also be involved (*10*).

The inadequacy of the current in vitro antimicrobial susceptibility testing for detecting fluoroquinolone treatment failure in typhoid fever is apparent in this case. According to NCCLS guidelines, Enterobacteriaceae (including typhoid salmonellae) are susceptible to the MIC of <1 μg/mL of ciprofloxacin, while resistant to the MIC of >4 μg/mL (*7*). But in our case-patient, treatment failed, even though the MIC was stated as 0.5 μg/mL. Similar observations have been made in other countries (*2*–*5*,*11*). Keeping in view this absence of correlation between MIC of fluoroquinolones and therapeutic response in typhoid fever, we recommended break point MIC values of ciprofloxacin in cases of typhoid salmonellae infection as follows: <0.125 μg/mL as susceptible, 0.125 μg/mL–1 μg/mL as reduced susceptibility, and >1 μg/mL as resistant. Determination of MIC may not be practicable in routine laboratory practice, particularly outside of a reference laboratory in developing countries. Also, disk diffusion criteria with ciprofloxacin are inadequate to highlight these new recommended MICs, and detecting mutation in the QRDR of *gyrA* gene by polymerase chain reaction (PCR) would not be practical or cost-effective. Several authors have reported a correlation between resistance to nalidixic acid and reduced susceptibility to ciprofloxacin and other fluoroquinolones (*2*,*11*). Routine testing of resistance to nalidixic acid with a disk content of 30 μg can serve as a useful screening test for fluoroquinolone resistance (*11*). However, revision of the diagnostic criteria for detecting fluoroquinolone resistance in typhoid salmonellae is needed, particularly to validate clinically all the laboratory-based anecdotal studies. Even with adoption of the new recommended MICs of fluoroquinolones against typhoid salmonellae, MICs would have to be correlated with inhibition zone size by disk diffusion technique and the clinical response in infection with typhoid salmonellae depicting reduced susceptibility against quinolones.

To summarize, fluoroquinolones are the most effective antimicrobial agents for treating enteric fevers (*1*). Emergence of resistance against them is of major concern. The spread of this resistance would leave only the less effective (*1*,*2*), but more expensive, third-generation cephalosporins for treatment of typhoid. Fluoroquinolone resistance must be identified early, and these drugs must be used judiciously. Otherwise, society may be faced with the prospect of untreatable typhoid fever.
